# Metabolic profile and hepatoprotective effect of *Aeschynomene elaphroxylon* (Guill. & Perr.)

**DOI:** 10.1371/journal.pone.0210576

**Published:** 2019-01-10

**Authors:** Mona M. Hashem, Maha M. Salama, Faten F. Mohammed, Adel F. Tohamy, Kadriya S. El Deeb

**Affiliations:** 1 Department of Pharmacognosy, Faculty of Pharmacy, Cairo University, Giza, Egypt; 2 Department of Pharmacognosy, Faculty of Pharmacy, The British University in Egypt, El-Sherouk City, Egypt; 3 Department of Pathology, Faculty of Veterinary Medicine, Cairo University, Giza, Egypt; 4 Department of Toxicology and Forensic medicine, Faculty of Veterinary Medicine, Cairo University, Giza, Egypt; 5 Institute of Pharmacology, Toxicology and Pharmacy, University of Veterinary Medicine Hannover, Hannover, Germany; Jadavpur University, INDIA

## Abstract

Liver diseases are life-threatening and need urgent medical treatments. Conventional treatment is expensive and toxic, so the urge for nutraceutical hepatoprotective agents is crucial. This study is considered the first metabolic profile of *Aeschynomene elaphroxylon* (Guill. & Perr.) extracts of; flowers, leaves & bark adopting UPLC-Orbitrap HRMS analysis to determine their bioactive metabolites, and it was designed to investigate the potential hepatoprotective activity of *A*. *elaphroxylon* flowers and bark extracts against CCl_4_-induced hepatic fibrosis in rats. Forty-nine compounds of various classes were detected in the three extracts, with triterpenoid saponins as the major detected metabolite. Flowers and bark extracts presented similar chemical profile while leaves extract was quite different. The antioxidant activities of the flowers, leaves & bark extracts were measured by *in vitro* assays as Fe^+3^ reducing antioxidant power and Oxygen radical absorbance capacity. It revealed that flowers and bark extracts had relatively high antioxidant activity as compared to leaves extract. Based on the metabolic profile and *in vitro* antioxidant activity, flowers and bark ethanolic extracts were chosen for alleviation of hepatotoxicity induced by CCl_4_ in rats. The hepatoprotective activity was studied through measuring hepatotoxicity biomarkers in serum (ALT, AST, and Albumin). Liver tissues were examined histopathologically and their homogenates were used in determining the intracellular levels of oxidative stress biomarkers (MDA, GSH), inflammatory markers (TNF-α). Flowers and bark ethanolic extracts exerted a significant hepatoprotective effect through reduction in the activities of ALT, AST and Albumin, the tested extracts reduced oxidative stress by increasing GSH content and reducing the MDA level. Furthermore, the extracts decreased levels of pro-inflammatory TNF-α. Moreover, the present study revealed the potentiality of *A*. *elaphroxylon* in ameliorating the CCl_4_-induced hepatic fibrosis in rats. In this aspect, *A*. *elaphroxylon* can be used with other agents as a complementary drug.

## Introduction

Liver is the main organ regulating homeostasis in the body, where metabolism and detoxification take place. It is the organ mostly involved in all the biochemical pathways related to growth, immunity, nutrient supply, provision of energy. Therefore, the liver is susceptible to injury which might occur by chronic exposure to drugs; such as paracetamol and acetaminophen, environmental toxicants and other xenobiotics as carbon tetrachloride (CCl_4_) and thioacetamide (TAA) [1,2].

Hepatotoxins such as carbon tetrachloride (CCl_4_), a widely-known mediator used in animal-models of hepatotoxicity, is metabolized by microsomal cytochrome (CYP) P450 enzymes into highly reactive metabolites as trichloromethyl (-CCl_3_) and trichloromethyl peroxy (-CCl_3_OO) radicals, which activate Kupffer cells, initiate the hepatic inflammation process where proinflammatory mediators such as tumor necrosis factor-a (TNF-α), interleukin-1β (IL-1β), and interleukin-6 (IL-6) are generated and induces lipid peroxidation [3]. CCl_4_ model in rodent has been extensively used to evaluate the efficacy of hepatoprotective drugs since this model can definitely reflect the metabolic and morphological changes that occur in liver injury with highly reproducibility [4–6].

Despite of the great scientific achievement in the field of hepatology, management of liver diseases is still a challenge to modern medicine. Natural products are known to be used as alternative remedies in the treatment of liver toxicity. Silymarin (Si) is an herbal drug used almost in liver protection all over the world. It exerts its effect through antioxidation, anti-lipid peroxidation, antifibrotic, anti-inflammatory, membrane stabilizing, immunomodulatory and liver regenerating mechanisms [7]. Several studies have publicized that hepatoprotective effects are related to phytoextracts rich in natural triterpenoidal saponins [8,9] such as Soyasaponins; that possesses a protective effect on liver injury [10,11].

*Aeschynomene elaphroxylon* (Guill. & Perr.) is large shrub or small tree up to 9–12 m tall, belonging to the family Fabaceae. It is native to the tropical Africa and Madagascar, cultivated in Egypt, Java and South America [12], Plants of genus *Aeschynomene* are reported to exert hepatoprotective [13], antimicrobial [14], cytotoxic [15], antioxidant [16], anthelmintic [17] and anti-inflammatory activities [18]. Preliminary phytochemical studies among different *Aeschynomene* species revealed the presence of flavonoid glycosides [19], pterocarpans [20] and chalcones [21]. On other hand, only some kaempferol derivatives were isolated from *A*. *elaphroxylon* aerial parts [22].

Based on presumed therapeutic value of genus *Aeschynomene* and the scarcity of reports concerning chemical and biological investigation of *Aeschynomene elaphroxylon*. This study focuses on two aspects: i) to perform comprehensive metabolic profiling of the active constituents of flowers, bark and leaves extracts of the *A*. *elaphroxylon* growing in Egypt using UPLC-Orbitrap-HRMS technique ii) to determine the hepatoprotective, anti-inflammatory and antioxidant effects of *A*. *elaphroxylon* (Guill. & Perr.) ethanolic extracts of flowers and bark against CCl_4_-induced liver hepatotoxicity in rats compared to conventional hepatoprotective drug ‘silymarin’.

## Materials and methods

### Chemicals and reagents

Silymarin (reference hepatoprotective), Carbon tetrachloride (CCl_4_), trichloroacetic acid (TCA), 2,2¨ -azobis (2-amidinopropane) dihydrochloride (AAPH), fluorescein disodium salt, [(±)-6-hydroxyl-2,5,7,8-tetramethlychromane-2-carboxylic acid] (Trolox), Gallic acid and thiobarbituric acid were purchased from Sigma-Aldrich Chemicals Co. (St. Louis, MO, USA). Solvents were of LC-MS grade (Sigma-Aldrich, Germany). All other chemicals used were of highest analytical grade.

### Plant materials

*Aeschynomene elaphroxylon* (Guill. & Perr.) flowers, bark and leaves samples were collected during March-April 2016 from Orman Botanic Garden, Giza, Egypt. No permits were required for the described study, which complied with all relevant regulations; there is an agreement between the ministry of the agriculture & Cairo University, that researchers have access to undergo their researches on the medicinal plants that are growing in this garden without endangering the genus or species. After authentication of the plant at the herbarium of the Orman Botanic garden by botanist and consultant at Orman Botanical garden, Giza, Egypt; Dr. Mohamed Gibali, the researches get his/her approval from the department council & faculty council to take the required samples for his/her study. A voucher specimen (20.4.16) was deposited at the herbarium of the Department of Pharmacognosy, Faculty of Pharmacy, Cairo University. Flowers (250 g), leaves (2 kg) and dried bark (2 g) were separately shade dried at room temperature for 10 days, powdered mechanically (25 000 rpm, 1 min) with herbs grinder to mesh size 1 mm.

### Preparation of the plant extracts

Air-dried powdered flowers (100 g), leaves (1 kg) and dried bark (1 Kg) were separately extracted by cold maceration in 70% ethanol till exhaustion. The combined extracts were filtered, and the supernatant was concentrated to dryness with a rotatory evaporator under reduced pressure at temperature not exceed 55°C. The obtained extracts were stored in air-tight desiccator to be used in the pharmacological studies.

### Sample preparation for UPLC-Orbitrap- HRMS analysis

Extraction of *A*. *elaphroxylon* (Guill. & Perr.) speciemens was carried out according to the protocol defined in Farag *et al* [23]. Dried and deep-frozen flowers, leaves and bark were powdered with pestle and mortar using liquid nitrogen. Flowers, leaves and bark powders (120 mg each) were mixed with 5ml 100% methanol (MeOH) containing 10 μg/ml umbelliferone as internal standard, using a Turrax mixer (11,000 rpm) for five 20-s periods with 1 min intervals separating each period to prevent heating, Then the extracts were vortexed vigorously and centrifuged at 3000 rpm for 30 min to remove debris and filtered using 22 μm pore size filter. An aliquot of 500 μl was placed on a (500 mg) C_18_ cartridge preconditioned with MeOH and H_2_O. Samples were then eluted using 5ml 100% MeOH, the eluent was evaporated under a nitrogen stream, and the collected dry residue was resuspended in 500 μl MeOH. Three microlitres of the supernatant was used for UPLC-MS analysis.

### UPLC-Orbitrap- HRMS analysis

Both negative and positive high resolution ESI modes and collision induced dissociation (CID) MS^n^ spectra were obtained from an Orbitrap Elite mass spectrometer (Thermo Fischer Scientific, Darmstadt, Germany) equipped with a heated electronspray ion source adjusted at 3 kV and 4 kV in negative and positive modes, respectively, capillary voltage of 300°C, source heater temperature of 250°C, FTMS resolution of 30.000. The MS spectrometer was coupled to an UHPLC system (Dionex UltiMate 3000, Thermo Fischer Scientific), equipped with a RP-18 column (30 mm × 2.1 mm × 1.8 μm), Acquity HSS T3, H_2_O, column temperature of 40°C. DAD (220–600 nm, Thermo Fischer Scientific). Mobile phase consisted of H_2_O (A) and acetonitrile (B) provided with 0.1% formic acid [24,25]. The following gradient elution was used: at 0–1 min 5% (B), followed by linear increase to reach 100% B till reach 11 min; then from 11 to 19 min (B) 100% was used, finally from 19 to 30 min (B) was reduced to be 5%. The flow rate used was 150 μl/min and the injection volume was 2 μl. The CID mass spectra were recorded using normalized collision energy (NCE) of 35%. Calibration of the instrument was operated externally by the using of Pierce ESI negative ion calibration solution (Product no. 88324) and Pierce ESI positive ion calibration solution (Product no. 88323) from Thermo Fisher Scientific. Evaluation of the data was done by using the software Xcalibur 2.2 SP1.

### Biological study

#### Animal maintenance

The study was performed on male Wistar albino rats (n = 25), weighing (200 ± 20 g, rats were obtained from the animal house of VACSERA Company (Giza, Egypt). This study was performed under authorized recommendations in the Guide for the Care and Use of Laboratory Animals of the College of Pharmacy, Cairo University. The protocol was approved by the Medical Research Ethics Committee (MREC), Faculty of Pharmacy, Cairo University (Approval ID: MP 1403). Rats were housed in small, clean polypropylene cages in an environmentally controlled room (23±2°C and 55 ± 5% humidity) and subjected to artificial light cycle with 12 hours light: dark cycle every day. Rats were maintained for one week to acclimate on food and water *ad libitum*. Rats were randomly divided into five groups (*n* = 5) and fed on the same diet throughout the experimental period (8 weeks).

#### *In vitro* antioxidant assays

**Fe^+3^ reducing antioxidant power assay.** The reducing power was determined for both flowers and bark extracts by using method described in Cai *et al*. [26]. The capacity of the sample to reduce Fe ^+3^ to Fe^+2^ in converting ferricyanide to ferrocyanide, whereby the yellow color of sample is turned to different shades of green or blue color (Perl's Purssian blue color) after addition of ferric chloride. The intensity of the formed color is measured spectrophotometrically depending on the reducing power of the tested samples. Briefly, samples (25 μL) of each extract at various concentrations (0.25–2.5 mg/ml in 50% methanol), gallic acid or methanol are mixed with (50 μl) of 50 μM Na2HPO4/KH2PO4 buffer (PH 6.6) and (50 μl) of 0.1% (W/V) K3Fe(CN)6, then incubated in water bath at 50°C for 20 min. Trichloroacetic acid solution (100 μl) of 1% (W/V) was added to the mixture and centrifuged at 3000 rpm for 10 min. The upper layer (250 μl) is carefully removed and mixed with (250 μl) of 5mM FeCl_3_ solution and then the absorbance of the developed color was measured spectrophotometrically after 10 min at 710nm using (tecan infinite F50 absorbance microplate reader). The assay was performed in a 96-well flat-bottom microplate. Where, the increased absorbance reading indicated increased reducing power. Ascorbic acid was used as positive control prepared in a concentration range of (0.5–10 mg/ml). All the tests were performed in triplicate. The relative reducing power of the samples was calculated; as compared to standard from the following formula:

Fe^+3^ reducing antioxidant power % = (1−*AS* /*AC*) X100

Where *AS* is the absorbance of sample and *AC* is the absorbance of standard at maximum concentration tested [27].

**Oxygen radical absorbance capacity (ORAC) assay.** The antioxidant activity of both flowers and bark extracts was determined using oxygen radical absorbance capacity (ORAC) assay described by Liang *et al*. [28]. In brief, 25 μl of each flowers and bark extracts at different dilutions, Trolox standard in concentration range (range 0.78–25 μM), or methanol prepared with 10 mM phosphate buffer (pH 7.4) were added to triplicate wells in a black, clear-bottom, 96-well microplate (Corning Scientific), and incubate at 37°C for 10 min. The outside wells of the plate were not used as there was much more variation from them than from the inner wells. 150 μL of 0.96 mM fluorescein (final concentration 2.5 nM) in phosphate buffer (pH 7.4) was added to each well and incubated at 37°C for 20 min. After this time, fluorescence measurements (excitation of 485 nm, emission of 520 nm) were taken every 90 s; first to determine the background signal. Afterward three cycles 25μl AAPH (final concentration: 60 mM) were added manually in each well with a multi-channel-pipette. This was done as quickly as possible as the ROS generator displays immediate activity after addition. Fluorescence measurements were continued for 90 min. Half life time of fluorescein was determined using MS Excel software.

#### *In vivo* hepatoprotective activity

**Determination of acute toxicity (LD_50_).** Median lethal dose (LD_50_) was determined for evaluating the safety of *A*. *elaphroxlon* Guill. & Perr. According to a procedure reported by Andress [29]. LD_50_ was estimated on five group of male albino mice (6 animal each, 25–30 g) for each extract, by oral treatment of single doses of flowers and stem bark ehanolic extracts separately (ranging from 1000–4000 mg/Kg b.wt.). No signs of toxicity or mortality were observed in any group within 30 min and then for 2, 4,8,24 and 48 hr after oral administration of plant extracts. Therefore, *A*. *elaphroxlon* (Guill. & Perr.) Flowers and stem bark extracts were considered safe up to 4000 mg/Kg b.wt. It is estimated that the therapeutic doses would be 1/20^th^ of the maximum dose was considered for *in vivo* studies.

**Experimental protocol.** Male Wistar albino rats (n = 25), were randomly divided into five groups (n = 5) and a following treatments were completed in 8 consecutive weeks. Group I, normal control, was administered 1% tween 80 orally three times weekly and corn oil intraperitoneally (IP) twice weekly. Group II (CCl_4_), the hepatotoxic group, received an IP injection of CCl_4_ and corn oil (1:1 v/v) mixture at dose of 0.5 ml/kg, twice per week. Group III (Silymarin Si+ CCl_4_), was daily treated with silymarin intragastrically in a dose of 100 mg/kg for 5 days/week for eight successive weeks and an IP injection of CCl_4_ and corn oil (1:1 v/v) mixture at dose of 0.5 ml/kg, twice per week [5]. Group IV was daily treated orally with ethanol (70%) extract of flower (F-Et) at doses 200 mg/kg BW then after 1 h received an IP injection of CCl_4_ and corn oil (1:1 v/v) mixture at dose of 0.5 ml/kg, twice per week. Group V was daily treated orally with ethanol (70%) extract of stem bark (B-Et) at doses 200 mg/kg BW then then after 1 h received an IP injection of CCl_4_ and corn oil (1:1 v/v) mixture at dose of 0.5 ml/kg, twice per week. The doses of flower F-Et and bark B-Et ethanol (70%) extracts were determined based on a preliminary experiment and was consistent with those in the reported literature [13]. Hepatotoxicity was confirmed during administration of CCl_4_ in live animals by taking blood samples at different time intervals from each group and measuring liver enzymes, ALT, AST and Albumin to ensure that they are elevated compared to normal group. Blood samples approximate (1ml) from each rat were drained from the retro-orbital plexus under anesthesia, and serum was obtained by vortexes vigorously and centrifuged at 4000 rpm for 8 min. The rats were sacrificed under anesthesia under proper anesthesia (2% ether), 24 h later last dose. Their livers were immediately dissected out, weighed, and cut into two parts. A part of the liver was mixed with phosphate buffer saline (PBS), another part was kept in formalin (10%) for histopathological and immunohistochemical examinations. After dissection the rats were frozen until incineration for hygienic disposal according to Faculty of Pharmacy, Cairo university waste disposal system.

#### Biochemical analysis

Biochemical analyses were carried out on all animals, serum ALT, AST and albumin (ALB) levels were measured colorimetrically using suitable commercial kits obtained from Spectrum Diagnostics (Al-Obour City, Cairo, Egypt) [30,31].

#### Oxidative stress marker determination

Lipid peroxidation, expressed as malondialdehyde (MDA) formation was assessed by measuring the concentration of thiobarbituric acid reactive substances calculated as malondialdehyde (MDA) in homogenates [32]. Reduced glutathione (GSH) levels in liver homogenate was determined using commercial kits (Biodiagnostics, Cairo, Egypt) [33]

#### Assessment of inflammatory response

Tumor necrosis factor alpha (TNF-α) expression was determined using ELISA kit (Assaypro Co., St. Charles, MO, USA).

#### Histopathological and immunohistochemical examinations

Representative liver specimens from each group were collected and fixed in 10% neutral buffered formalin. The specimen was routinely processed, and paraffin embedded then sliced into 5 μm thick sections on positively charged glass slides and stained by hematoxyline and eosin (H&E) and Masson`s Trichrom (MTC). Fibrosis was graded according to korb *et al*. [34] into six grades. The percentage of connective tissue deposition based on the blue staining of collagen by MTC at 10x magnification power field was performed via color deconvolution image J software.

For the immunohistochemical examination, three paraffin sections were deparaffinized by xylene for 15 minutes, rehydration in graded ethanol, blockage of endogenous perioxidase was performed by adding few drops of 0.3% hydrogen peroxides in absolute MeOH for 30 min. The sections were incubated in 5% skim milk for 30 min at room temperature. Antigen retrieval was done by heating in microwave at 500 W for 10 min with addition of 10 mM citrate buffer, pH 6.0 over the slide and slides were placed in the microwave. Sections were incubated overnight at 4°C in a humidified chamber with one of the following primary antibodies: mouse monoclonal antibody to *α-*SMA diluted 1:100 (mouse anti-SM-α actin, clone (DAKO). Anti-mouse IgG in rabbit (cat no. M7023; 1:500; Sigma-Aldrich) was used as the secondary antibody. The sections were washed with PBS, then incubated with Streptavidin peroxidase (Thermo Scientific). Slides were incubated for 10 min with 3, 3′-diaminobenzidine tetrahydrochloride (DAB, Sigma). Finally, the slides were counterstained with haematoxylin then dehydrated and mounted. The cells that were stained brown in the cytoplasm/nucleus were positive. Caspase-3 as an apoptotic marker using avidin-biotin Peroxidase (DAB, Sigma Chemical Co.). Tissue sections were incubated with a monoclonal antibody to caspase-3, their expression was localized by the chromogen 3,3-diaminobenzidine tetrahydrochloride (DAB, Sigma-Aldrich). The intensity of brown color staining was estimated by color deconvolution image J software.

#### Statistical analysis

Statistical analyses were carried out using GraphPad Prism v7 software. Data were expressed as mean ± SEM. Hypothesis testing methods included one-way analysis of variance (ANOVA) followed by *Tukey’s post hoc* test to determine the differences among the mean values of different groups. *P* < 0.05 was considered to indicate statistical significance.

## Results

### Identification of metabolites via UPLC-Orbitrap-HRMS

Flowers, bark and leaves extracts were analyzed using modern UPLC-orbitrap-HRMS operated in both positive and negative ESI modes. Metabolites of the extract were tentatively defined based on their accurate mass, distribution of their isotope, UV/V-is spectrum (200–600 nm), and fragmentation pattern and with comparison to phytochemical dictionary of natural products database (CRC) as well as comparison with reported literature.

**Fig 1:**
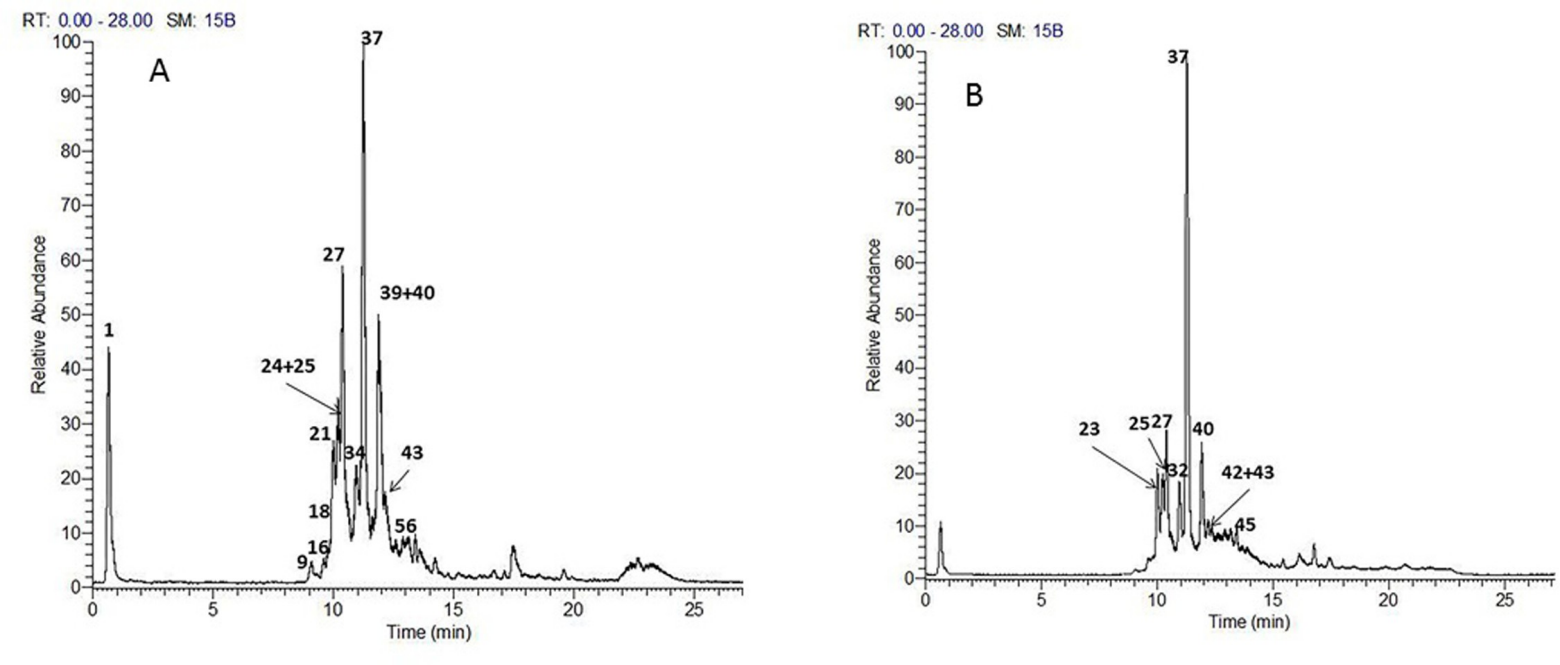
**TIC chromatograms of UPLC-Orbitrap-HRMS of *A*.*elaphroxylon* flower extract in both negative (A) and positive (B) ion modes.** Numbers refer to identified compounds listed in Table 1.

**Fig 2:**
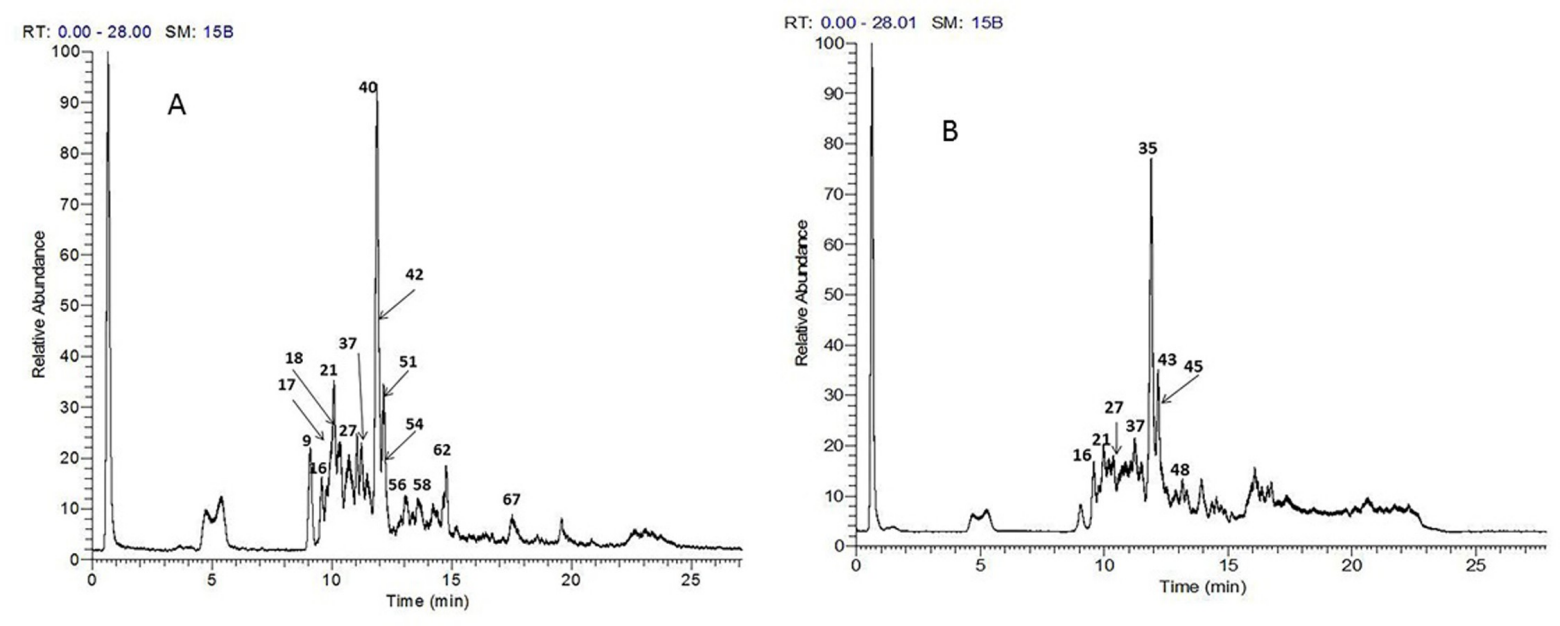
**TIC chromatograms of UPLC-Orbitrap-HRMS of *A*.*elaphroxylon* bark extract in both negative (A) and positive (B) ion modes.** Numbers refer to identified compounds listed in Table 1.

**Fig 3:**
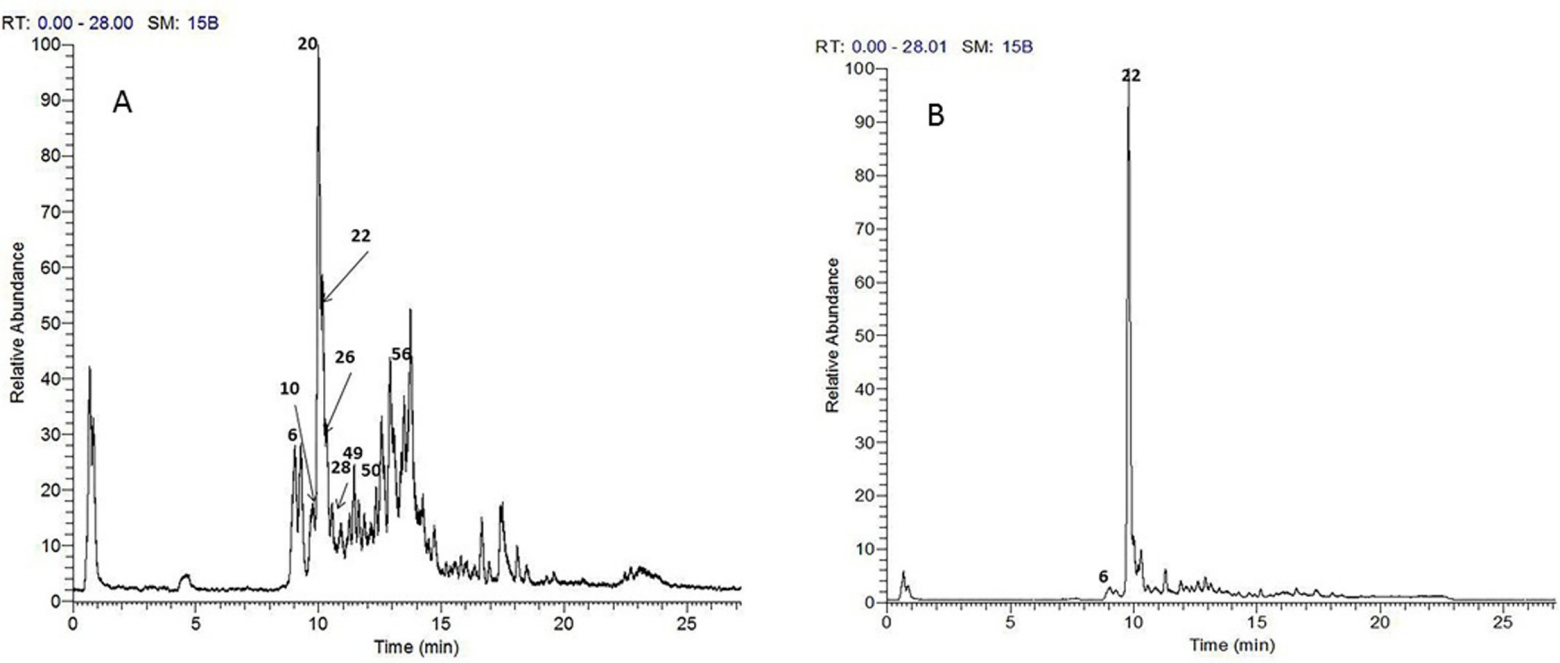
**TIC chromatograms of UPLC-Orbitrap-HRMS of *A*.*elaphroxylon* leaf extract in both negative (A) and positive (B) ion modes.** Numbers refer to identified compounds listed in Table 1.

The analytical method applied enabled the identification of 49 compounds, their structures were assigned as nine triterpenoid saponins, eleven phenolic acid derivatives, one anthocyanin, three proanthocyanins, thirteen flavonoid glycosides and twelve fatty acids. The identified compounds are listed in Table 1 and the chromatograms of *A*. *elaphroxylon* flower, stem bark and leaf extract in positive and negative ion mode were depicted in Figs 1, 2 and 3.

**Table 1:** Metabolites identified in *A*. *elaphroxylon* (Guill. & Perr.) flowers, bark and leaves extracts using UPLC- Orbitrap HRMS.

Peak no.	m/z [M-H]^-^	Mode	t_R_ (min)	λmax (nm)	Deprotonated moleclar Formula	Error (PPM)	MS/MS^−^	Identification	Plant organ		
									flower	bark	leaf
**Phenolic acids**											
1	191.0556	N	0.66	249	C_7_H_11_O_6_	0.635	173,127,111	Quinic acid	+	_	_
2	153.0192	N	1.94	262, 297	C_7_H_5_O_4_	5.5	109	Protocatechuic acid	+	_	_
3	315.0688	N	2.52	Nd	C_13_H_15_O_9_	6.5	169, 151	Gallic acid deoxysugar	+	_	_
4	255.0500	N	4.70	Nd	C_11_H_11_O_7_	0.51	237,195	Unidentified	+	+	-
5	337.0910	N	6.67	Nd	C_16_H_17_O_8_	2.088	191,163,173	5*- p-*coumaroylquinic acid	+	_	+
6	353.0866	N,P	8.81	280	C_15_H_17_O_8_	0.194	191,179	Chlorogenic acid	_	_	+
7	325.0916	N	8.91	Nd	C_11_H_11_O_6_		163, 145,119	*p-*Coumaroyl *-O-* hexose derivative	+	_	_
8	443.1914	N	8.97	Nd	C_21_H_31_O_10_	0.437	347,283,248	Unidentified	+	-	-
9	239.0554	N	9.21	Nd	C_16_H_19_O_9_	1.78	221,195,179, 149	Caffeic acid derivative	+	+	_
10	355.1026	N	9.55	215, 273	C_9_H_7_O_3_	0.680	217,193.175	feruloyl *-O-* hexoside	_	_	+
11	163.0399	N	9.91	216, 310	C_11_H_11_O_5_	6.068	119	*P-*Coumaric acid	_	_	+
12	431.1907	N	9.82	282	C_20_H_31_O_10_	0.206	385,225,179	Unidentified	_	+	_
13	223.0606	N	10.22	Nd	C_18_H_15_O_8_	0.540	205,179,163, 133	Sinapinic acid	_	+	_
14	359.0760	N	10.68	215, 273	C_15_H_17_O_8_	0.261	161,179,197	Rosmarinic acid	_	_	+
**Anthocyanins**											
15	321.0596	N	8.63	Nd	C_15_H_13_O_8_	1.23	277,233,284	Leucodelphinidin	+	_	_
**Proanthocyanidins**											
16	577.133	N, P	9.4	282, 334	C_30_H_25_O_12_	0.767	289,407,425, 451, 459	Procyanidin dimmer B1	+	+	_
17	289.0710	N, P	9.71	282, 321	C_15_H_13_O_6_	1.85	245,205, 179	(+)- Catechin (A)	+	+	_
18	865.1967	N	9.84	282, 321	C_54_H_37_O_18_	1.64	847,695,577, 575, 407	Procyanidin trimmer	_	+	_
**Flavonoid glycosides**											
19	595.1641	N, P	9.36	290, 314	C_27_H_31_O_15_	0.039	577,505, 475,329,359	Dihydrokaempferol -*C-*hexoside *-O-*rhamnoside	+	_	_
20	609.144	N	9.70	282, 318	C_27_H_29_O_16_	0.084	447,285	Kaempferol *-O-* di-hexoside	+	_	+
21	579.1703	N, P	9.91	284, 330	C_27_H_31_O_14_	0.314	561,489.459, 313, 343	Naringenin -*C-*hexoside *-O-*rhamnoside	+	+	_
22	625.1394	N, P	9.93	325	C_27_H_29_O_17_	0.633	607,581,505	Quercetin *-O-*hexoside *-C-* hexoside	_	_	+
23	331.0928	N, P	9.99	282	C_21_H_15_O_4_	10.82	313,287,269	Unidentified	+	_	_
24	593.149	N	10.13	271, 341	C_27_H_29_O_15_	0.534	447, 431,285	Kaempferol-3*-O*-rutinoside	+	_	_
25	563.139	N, P	10.21	274, 345	C_26_H_27_O_14_	0.234	447, 431,285	Kaemferol *-O-*deoxyhexoside pentoside	+	_	_
26	463.087	N	10.33	265, 303, 325	C_21_H_19_O_12_	0.189	301	Quercetin-3*-O-*hexoside	_	_	+
27	577.156	N, P	10.34	267, 344,	C_27_H_29_O_14_	1.128	431,417, 285	kaempferol-3,7*-O-α-*dirhamnoside	+	+	_
28	447.092	N	10.48	281, 321	C_21_H_19_O_11_	0.139	431, 285	Kaempferol *-O-* hexoside	+	+	+
29	417.081	N	10.68	281	C_20_H_17_O_10_	0.703	285	Kaempferol *-O-* pentoside	+	_	_
30	655.3054	N	10.72	216, 280	C_43_H_43_O_6_	0.065	632	Unidentified	_	+	_
31	431.097	N	10.81	282	C_21_H_19_O_10_	0.031	285	Kaemferol *-O-* deoxyhexoside	+	_	_
32	379.1657	N, P	10.84	215, 286	C_27_H_23_O_2_	9.59	259	Unidentified	+	_	_
33	467.2428	N	10.90	220	C_28_H_35_O_6_	0.032	452,449,423	Unidentified	_	_	+
34	725.206	N, P	10.98	284	C_36_H_37_O_16_	1.395	707,635,605, 579, 563, 272	Naringenin *-C-*hexoside *-O-*rhamnoside cinnammoyl derivative	+	_	_
35	582.2598	N, P	11.1	292	C_28_H_40_O_12_N	9.14	462,436, 342,372	Quercetin *-C-*hexoside *-O-*rhamnoside derivative	+	_	_
36	531.2584	N	11.21	221	C_29_H_39_O_9_	0.714	513,485,467	Unidentified	_	_	_
**Triterpenoid saponins**											
37	957.5037	N, P	11.17	220, 281	C_48_H_77_O_19_	1.636	939, 895, 811, 767, 631,473	Soyasaponin V	+	+	_
38	955.4880	N	11.51	221, 282	C_48_H_75_O_19_	1.666	937, 911, 893, 809, 539, 471	deoxyhexose -hexose-hexuronic acid Hederagenin	_	+	_
39	911.4979	N	11.80	222	C_47_H_76_O_18_	1.609	893,849,765, 615, 571,457	Soyasaponin II	+	_	_
40	941.5081	N, P	11.85	222	C_48_H_77_O_18_	2.466	923, 879, 795, 751, 633, 457	Soyasaponin I	+	+	_
41	925.5143	N	11.94	222	C_48_H_77_O_17_	1.283	907, 863,779, 599.509,439	Unknown teriterpenoid saponin	+	_	_
42	795.4508	N, P	11.97	222	C_42_H_67_O_14_	1.723	733, 633, 525, 457	Soyasaponin III	_	+	_
43	939.4924	N, P	12.1	222	C_48_H_75_O_18_	2.45	921, 877, 793, 613, 523, 455	Pisumsaponin II	+	+	_
44	895.5026	N, P	12.17	222	C_47_H_75_O_16_	2.538	877, 833, 749, 731, 599, 439	Unknown teriterpenoid saponin	+	_	_
45	1067.539	N, P	12.17	222	C_54_H_85_O_21_	0.182	1049, 921, 759, 741, 651, 457	Soyasaponin *βg*	_	+	_
46	923.4980	N	12.24	222	C_48_H_75_O_17_	1.978	905, 861, 777, 597, 507, 437	Unknown teriterpenoid saponin	+	_	_
47	633.3981	N	12.29	222	C_36_H_57_O_9_	2.494	615, 457	Hexuronic acid Soyasapogenol B	_	+	_
48	921.4824	N, P	12.30	222	C_48_H_73_O_17_	1.747	741, 457	Soyasaponin *γg*	_	+	_
**Fatty acids**											
49	327.2167	N	11.52	220, 274	C_18_H_31_O_5_	0.058	291, 229, 211,183	Trihydroxy-octadecadienoic acid (C_18:2_)	+	+	+
50	329.2320	N	11.67	220, 276	C_18_H_33_O_5_	0.787		Trihydroxy-octadecaenoic acid (C_18:1_)	+	+	+
51	287.2215	N	11.74	218	C_16_H_31_O_4_	0.089	311,229,211	Dihydroxy-hexadecanoic acid (C_16:0_)	+	_	_
52	609.2660	N	11.77	222	C_41_H_37_O_5_		429,293	Unidentified	_	_	+
53	309.2065	N	11.94	222	C_18_H_29_O_4_	1.630	248, 174, 161	Dihydroxy-octadecatrienoic acid	_	_	+
54	307.1909	N	11.97	222	C_18_H_27_O_4_	1.739	291,251,171	hydroxy-oxooctadeca-trienoic acid	_	_	+
55	653.2643	N	12.06	222	C_28_H_45_O_17_	1.203	607,565	Unidentified	_	_	+
56	311.222	N	12.28	223	C_18_H_31_O_4_	1.459	289,235,185	Octadec-2-enedioic acid (C_18:1_)	+	+	_
57	695.2748	N	12.41	222	C_30_H_47_O_18_	1.224	607,565	Unidentified	_	_	+
58	313.237	N	12.49	223	C_18_H_33_O_4_	1.449	293,275,223	Octadecanedioic acid (C_18:0_)	+	+	_
59	721.3631	N	12.50	222	C_34_H_57_O_16_	1.320	675	Unidentified	_	_	+
61	293.2116	N	12.92	220	C_18_H_29_O_3_	0.001	295,277,201	Hydroxy-octadecatrienoic acid (C_18:3_)	+	+	+
62	295.2268	N	13.15	220	C_18_H_31_O_3_	0.334	275,235,211,	Hydroxy-octadecadienoic acid (C_18:2_)	+	+	+
63	743.3468	N	13.53	222	C_36_H_55_O_16_	2.155	697,579,529	Unidentified	_	_	+
64	555.2826	N	13.63	224	C_28_H_43_O_11_	5.351	556.299.225	Unidentified	_	_	+
65	271.2273	N	14.23	220	C_16_H_31_O_3_	0.148	277,195,171	Hydroxy-hexadecanoic acid (C_16:0_)	+	_	_
66	455.3518	N	15.69	224	C_30_H_47_O_3_	1.49	437, 411,393	Oleanolic acid	+	_	_
67	341.2685	N	17.68	225	C_20_H_37_O_4_	0.21	313,269	Eicosanedioic acid	+	+	_

Nd. Not defined, N. negative, P. positive

#### Triterpenoid saponins

Triterpenoid saponins were detected for the first time in genus *Aeschynomene*. Saponins were represented by twelve compounds from peak 37 to 48, most of the detected saponins were soyasaponins. Peak 37 corresponds to deoxyhexose -hexose-hexuronic acid soyasapogenol B (Soyasaponin V), identified by an ion at *m/z* 957.50372 with a formula (C_48_H_77_O_19_)^-^, The MS^2^ of the compound was at *m/z* 939 (M-H_2_O-H)^-^, *m/z* 895 (M-H_2_O-CO_2_-H)^-^, *m/z* 811 (M- deoxyhexose-H)^-^, *m/z* 767 (M- deoxyhexose -CO_2_-H)^-^, *m/z* 749 (M- deoxyhexose—H_2_O-CO_2_-H)^-^, *m/z* 631 (M- deoxyhexose—Hexose—H_2_O-H)^-^, *m/z* 613 (M- deoxyhexose—Hexose—H_2_O- H_2_O-H)^-^, *m/z* 541 (M- deoxyhexose—hexose—H_2_O-108-H)^-^ and *m/z* 473 (aglycone-H)^-^ [35]. This saponin; soyasaponin V is the major detected triterpenoid saponin in the flowers, while soyasapogenol Bb (peak 40) is the major identified saponin in the bark, its MS2 exhibited an ion peak at *m/z* 923 (M-H_2_O-H)-, *m/z* 879 (M-H_2_O-CO_2_-H)-, *m/z* 795 (M-deoxyhexose-H)-, *m/z* 751(M- deoxyhexose -CO_2_-H)-, *m/z* 733 (M- deoxyhexose—H_2_O-CO_2_-H)-, *m/z* 633(M- deoxyhexose—hexose -H)-, *m/z* 615 (M- deoxyhexose -Glucose-H)-, *m/z* 597 (M- deoxyhexose—hexose—H_2_O- H_2_O-H)-, *m/z* 525 (M- deoxyhexose—Hexose—H_2_O-108-H)- and *m/z* 457 (Soyasapogenol B-H)-. Peak 39 with [M-H]- at *m/z* 911.4979 corresponding to the formula (C_48_H_75_O_17_)—was identified as (Soyasaponin II); deoxyhexose -pentose-hexuronic acid soyasapogenol B [36]. The MS^2^ of the compound was at *m/z* 893 (M-H_2_O-H)^-^, *m/z* 849 (M-H_2_O-CO_2_-H)^-^, *m/z* 765 (M-deoxyhexose-H)^-^, *m/z* 703 (M- deoxyhexose—H_2_O-CO_2_-H)^-^, *m/z* 615 (M- deoxyhexose—pentose—H_2_O-H)^-^, *m/z* 597 (M- deoxyhexose—pentose—H_2_O- H_2_O-H)^-^, *m/z* 571 (M- deoxyhexose–pentose-H_2_O-CO_2_-H)^-^, *m/z* 525 (M- deoxyhexose—pentose -108-H)^-^ and *m/z* 457 (Soyasapogenol B-H)^-^. Peak 42 showed a molecular ion at *m/z* 795.4508 with a formula (C_42_H_67_O_14_)—and a MS^2^ at *m/z* 733 (M-H_2_O-CO_2_-H)^-^, *m/z* 633 (M-hexose-H)^-^, *m/z* 615 (M-hexose-H_2_O-H)^-^, 525 (M-Hexose -108-H)^-^, *m/z* 457 (Soyasapogenol Bb-H)^-^, was identified as hexose-hexuronic acid soyasapogenol B (Soyasapogenol Bb') [37].

Peak 43 with [M-H]^-^ at *m/z* 939.49249 corresponding to the formula (C_48_H_75_O_18_)^-^ was identified as pisumsaponin II. The MS^2^ of the compound was at *m/z* 921 (M-H_2_O-H)^-^, *m/z* 877 (M-H_2_O-CO_2_-H)^-^, *m/z* 793 (M- deoxyhexose-H)^-^, *m/z* 731 (M- deoxyhexose—H_2_O-CO_2_-H)^-^, *m/z* 613 (M- deoxyhexose—hexose—H_2_O-H)^-^, *m/z* 595 (M- deoxyhexose—hexose—H_2_O- H_2_O-H)^-^, *m/z* 523 (M- deoxyhexose—hexose—H_2_O-108-H)^-^ and *m/z* 455 (Sandosapogenol-H)^-^ [38].

#### O/C-Flavonoid glycosides

Flavonoids were detected and represented by twelve peaks. Based on MS/MS and UV/V-is spectra, all the identified flavonoids were derivatives of quercetin, kaempferol and naringinin *O/C-*glycosides. Flavonoid glycosides were characterized by the presence of mass signals [M − H]^−^ at *m/z* 285 (C_15_H_9_O_6_^-^, kaempferol), 301 (C_15_H_9_O_7_^-^, quercetin) and 271 (C_15_H_12_O_5_^-^, naringinin). Kaempferol-3*-O-*rutinoside (Peak **24**) was identified from its mass fragmentation pattern. Fragment ion at *m/z* 447 indicating the loss of 146 amu (rhamnosyl moiety), *m/z* 431 indicating loss of (a hexose moiety) 162 amu [39,40]. Peak **27** corresponds to kaempferitrin (kaempferol 3, 7*-O-*α-dirhamnoside), identified by an ion at m/z 577.1549 with a molecular formula (C_27_H_29_O_14_)^-^ and fragment ions at *m/z* 431, indicating loss of 146 amu (rhamnosyl moiety) and a fragment of *m/z* 285, assigned to k aempferol aglycone with the loss of 146 amu (rhamnosyl moiety) [41]. Peak **20** was displayed with [M − H]^−^ at *m/z* 609.1445 (C_27_H_29_O_16_)^-^ and fragment ions in the MS^2^ spectrum appearing at *m/z* 447 [M-162-H] ^−^ and *m/z* 285 [M-162-H]^−^, this is in agreement with kaempferol di*-O-* hexoside.

Regarding C-glycosides, the fragmentation patterns were observed in peaks 19, 21, 34 and 35. The characteristic fragmentation pathways of C-glycosyl flavonoids shows dehydration (−18 amu) and cross-ring cleavages between [(*O-*C1 and C2-C3)] or [(*O-*C1 and C3-C4)] of the sugar units, viz., [M-120/ 90]^+/−^ for *C-*hexosides, [M-90/60]^+/−^ for *C-*pentosides, and [M-104/74]^+/−^ for *C-*deoxyhexosidescross-ring [42,43].

In *A*. *elaphroxylon*, mono*-C* and di*-C-*glycosides were identified as flavanone and flavanonol derivatives of naringenin and dihydrokaempferol respectively. The ions [Agl + 41/Agl + 71]− in mono-C and [Agl + 83/Agl + 113]− in di-C glycosides typify the aglycon (Agl) [43], The ESI-MS spectrum of Peak 19 exhibited a molecular ion (M-H)^-^ at *m/z* 595.1641 (C_27_H_31_O_15_)—was assigned to dihydrokaempferol -*C-*hexoside *-O-*rhamnoside from its product ions: *m/z* 577 [M-18-H] ^−^ (–H_2_O), *m/z* 505 [M-90-H] ^−^, *m/z* 475 [M-120-H]^−^, *m/z* 329 [Agl + 41]^−^ and *m/z* 359 [Agl + 71]^−^. This is in accordance with flavanonol mono*-C-*hexoside. Peak **21** exhibited a molecular ion [M − H]^−^ at *m/z* 579.1703 (C_27_H_31_O_14_)^-^ and its MS^2^ spectrum showed ions at *m/z* 561 [M-18-H]^−^ (–H_2_O), *m/z* 489 [M-90-H]^−^ and a base peak at *m/z* 459 [M-120-H]^−^, *m/z* 313 [Agl + 41]^−^ and *m/z* 343 [Agl + 71]^−^. This is a typical fragmentation pattern of flavanone mono*-C-*hexoside, Peak **21** was identified as naringenin -*C-*hexoside *-O-*rhamnoside. Peak 34 was characterized by a molecular ion peak [M − H]^−^ at *m/z* 725.2066 (C_36_H_37_O_16_)^-^ and its product ions at *m/z* 707 [M-18-H]^−^ (–H_2_O), *m/z* 635 [M-90-H]^−^, together with a base peak at *m/z* 605 [M-120-H]^-^, another at *m/z* 579 [M-146-H] ^−^ indicating the loss of rhamnosyl moiety, *m/z* 563 [M-162-H]^-^ corresponding to the loss of cinammoyl moiety and *m/z* 272 [M+H]^+^ in positive ion mode assigned to naringinin aglycone, this compound was regarded as naringenin *-C-*hexoside *-O-*rhamnoside cinnammoyl derivative. To the best of our knowledge, this is the first report for the presence of Flavonoid*-C-*glycosides in *Aeschynomene* species.

#### Proanthocyanidins

(Epi) catechin peak 17, (Epi) catechin dimmer peak 16 and (Epi) catechin trimmer peak 18 were detected in the first half of MS chromatograms. (Epi) catechin peak 17 exhibited a deprotonated molecular ion at *m/z* 289.0711 (C_15_H_13_O_6_)—and revealed the characteristic MS^2^ fragments at *m/z* 245 resulting from the loss of CO_2_ and *m/z* 205, 203 corresponding to the cleavage of the A-ring of flavan-3-ol. Peak 16 showed a molecular ion at *m/z* 577.1345 (C_30_H_25_O_12_)^-^ corresponded to procyanidin dimmers B (EC-EC), The retro-Diels-Alder rearrangement (RDA) fragmentation of the dimers produced mostly product ions at m/z 425 [M−H−152]^−^. The fragment ion at *m/z* 407 result resulted from water elimination [M−H−152−18]^−^ [44], another detected fragment ions at *m/z* 451 result from the elimination of the phloroglucinol molecule (A-ring) [M−H−126]− [45], and fragment ion result from the loss of an (epi)catechin molecule at *m/z* 289. Peak 18 [M−H]^−^at *m/z* 865.1959 with molecular formula (C_54_H_37_O_18_)^-^ was identified as procyanidin trimmers (EC-EC-EC), with MS^2^ fragment ions at *m/z* 713, with a loss of 152 amu, resulting from RDA fission of the heterocyclic ring system, the base peak ion at *m/z* 695 with the a loss of 170 amu, corresponds to the RDA fission with an additional loss of water. This was further confirmed by the product ion at *m/z* 577 [M−H−288]^-^ [46]. Ions at *m/z* 451 and *m/z* 407 supported the characteristic fragmentation pattern of the procyanidin dimers as described above.

#### Hydroxycinnamic acid derivatives

This study revealed the presence of several hydroxycinnamic acid derivatives, such as caffeic, ferulic-, *p-*coumaric acid, quinic acid and sinapinic acid. Peak 6 revealed [M−H]^−^ at *m/z* 353.08664 with molecular formula (C_16_H_17_O_9_)^-^ comparable to caffeoylquinic acids. This peak was identified as positional isomers of 5*-O-*caffeoylquinic acid. The predominant fragment at 191 amu for quinic acid in the MS^2^ spectrum and characteristic UVmax values at 298 and 327 nm are diagnostic for hydroxycinnamic acid derivatives [47].

#### Hydroxybenzoic acid derivatives

Minor peaks correspond to hydroxybenzoic acid derivatives such as gallic acid derivative, was represented by peak 3. Protocatechuic acid peak 2 produced the ions at *m/z* 125, *m/z* 123, *m/z* 109 and *m/z* 93, respectively due to loss of CO_2_ from their respective precursor ions [48].

#### Fatty acids

In the last section of the chromatographic run, negative ionization MS revealed the presence of several fatty acids in *A*. *elaphroxylon* different organs; flowers, bark and leaves extracts, Peaks 56 and 58 corresponding to saturated fatty acids, were identified as Octadec-2-enedioic acid and Octadecanedioic acid. Peaks 49 and 50 showed a mass weight of and 327.2167 and 329.2320 amu, with a molecular formulae (C_18_H_33_O_5_) ^−^ and (C_18_H_31_O_5_) ^−^, respectively. These were identified as trihydroxyoctadecadienoic acid and trihydroxyoctadecaenoic acid. Other oxygenated fatty acids were observed in peaks 51 and 65, showing a mass weight of 287.2215 and 271.2273 amu with predicted molecular formulae of (C_16_H_31_O_5_)^−^ and (C_16_H_31_O_4_)^−^, respectively. These were assigned as dihydroxyhexadecanoic and hydroxyhexadecaenoic acid. MS signal was also assigned for saturated fatty as eicosanedioic acid 67, as evident from high resolution mass 341.2685 with predicted molecular formulae of (C_20_H_37_O_4_) ^−^.

### *In vitro* antioxidant assays

#### Fe^+3^ reducing antioxidant power assay

One of the most significant antioxidant mechanism that reflect the antioxidant power is the reducing power [49]. FRAP test is considered a simple, reliable and reproducible method to measure the antioxidant capacity [50]. Whereby, compounds with reducing power are electron donors, and they can decrease the oxidized intermediates of lipid peroxidation processes, so they can act as primary and secondary antioxidants.

The IC_50_ values of FARP of flowers and bark extracts were (633.12 and 513.45 μg/ml).) respectively, which showed significant ferric chelating ability compared with IC_50_ of gallic acid (547.14 μg/ml).

#### Oxygen radical absorbance capacity (ORAC) assay

Oxygen radical absorbance capacity (ORAC) assay has been widely accepted as a tool to test the antioxidant activity where reactive oxygen species, ROS which are produced by thermal degradation of 2,2¨-azobis(2-amidinopropane) dihydrochloride (AAPH) and quench the signal of the fluorescent probe fluorescein. The subsequent addition of antioxidants reduces the quenching by preventing the oxidation of the fluorochrome. the antioxidant activity ED_50_ of flowers and bark extracts were with a value of (60.11±4.14 and 26.9±4.22 μg/mL). while, that of Trolox was 27.0 ± 12.37 μg/mL.

### Hepatotoxicity markers

Challenging rats with CCl_4_ significantly increased hepatocellular injury biomarkers (AST, ALT, and ALB) in serum as compared to normal control group are shown in Table 2. However, Pretreatment with silymarine, flowers and bark ethanolic extracts produced a significant decrease in liver enzyme levels as compared to CCl_4_ group. As represented from our results, the *A*. *elaphroxylon* shows a significant reduction in the activities of ALT and AST in addition to ALB concentration; flowers extracts (46, 31 and 26%, respectively) while bark extracts (48, 35 and 24%, respectively) compared to the CCl_4_-intoxicated group. This effect was comparable to that of silymarin at a dose of 100 mg/kg (53, 39 and 43%, respectively).

**Table 2:** Effect of *A*. *elaphroxylon* flowers and bark extracts and silymarin on liver functions, TNF-α, oxidative stress Markers against CCL_4_-induced hepatic damage.

	Normal	CCl_4_	Si + CCl_4_ (100mg /kg/d)	F-Et +CCl_4_ (200mg /kg/d)	B-Et +CCl_4_ (200mg /kg/d)
**ALT (U/ml)**	30.52±2.08^(^^a^^)^	79.26±9.59^(^^b^^)^	37.35±8.58^(^^a^^)^	43.23±3.65^(^^a^^)^	41.31±6.01^(^^a^^)^
**AST (U/ml)**	41.23±1.31^(^^a^^)^	78.81±0.72^(^^b^^)^	47.81±0.78^(^^a^^)^	54.45±0.57^(^^a^^)^	51.46±0.55^(^^a^^)^
**Albumin (g/dl)**	3.46±0.057^(^^a^^)^	2.35±0.067^(^^b^^)^	3.36±0.057^(^^a^^)^	3.15±0.11^(^^a^^)^	3.21 ± 0.46^(^^a^^)^
**GSH (mmol/g tissue)**	2.66 ± 0.23^(^^a^^)^	1.12 ± 0.15^(^^b^^)^	2.58 ± 0.14^(^^a^^)^	2.12 ± 0.04^(^^a^^)^	2.32±0.156^(^^a^^)^
**MDA (nmol/g tissue)**	6.31 ± 0.522^(^^a^^)^	12.44 ± 1.311^(^^b^^)^	8.72 ± 0.847^(^^a^^,^^b^^)^	8.41± 0.454^(^^a^^)^	7.40±0.771^(^^a^^)^
**TNF-α (ng/g tissue)**	0.30±0.029^(^^a^^)^	0.63±0.031^(^^a^^)^	0.35±0.034^(^^a^^)^	0.43±0.037^(^^a^^)^	0.46±0.035^(^^a^^)^

Si = silymarin, F-Et = Ethanol (70%) extract of flowers, B-Et = Ethanol (70%) extract of bark.

Each value expressed as mean ± SEM, (n = 5).

^(a)^ Significantly different from CCl_4_ group at *p* < 0.05.

^(b)^Significantly different from normal group at *p* < 0.05.

Statistical analysis was performed using one-way ANOVA followed by Tukey -Kramer as a post hoc test.

### Oxidative stress markers

The administration of silymarin (100 mg/kg) and both *A*. *elaphroxylon* flowers and bark ethanolic extracts (200 mg/kg) significantly increase in GSH content (130, 89 and 107%, respectively), and significantly reduced the MDA level (30, 32 and 41%, respectively) relative to the CCl_4_-intoxicated group as shown in Table 2.

### Anti-inflammatory activity study

The level of TNF-α was significantly decreased in the groups treated with both *A*. *elaphroxylon* flowers and bark ethanolic extracts and silymarin (32, 27 and 44%, respectively), compared to the CCl_4_-intoxicated rats shown in Table 2.

### Histopathological findings

**Fig 4:**
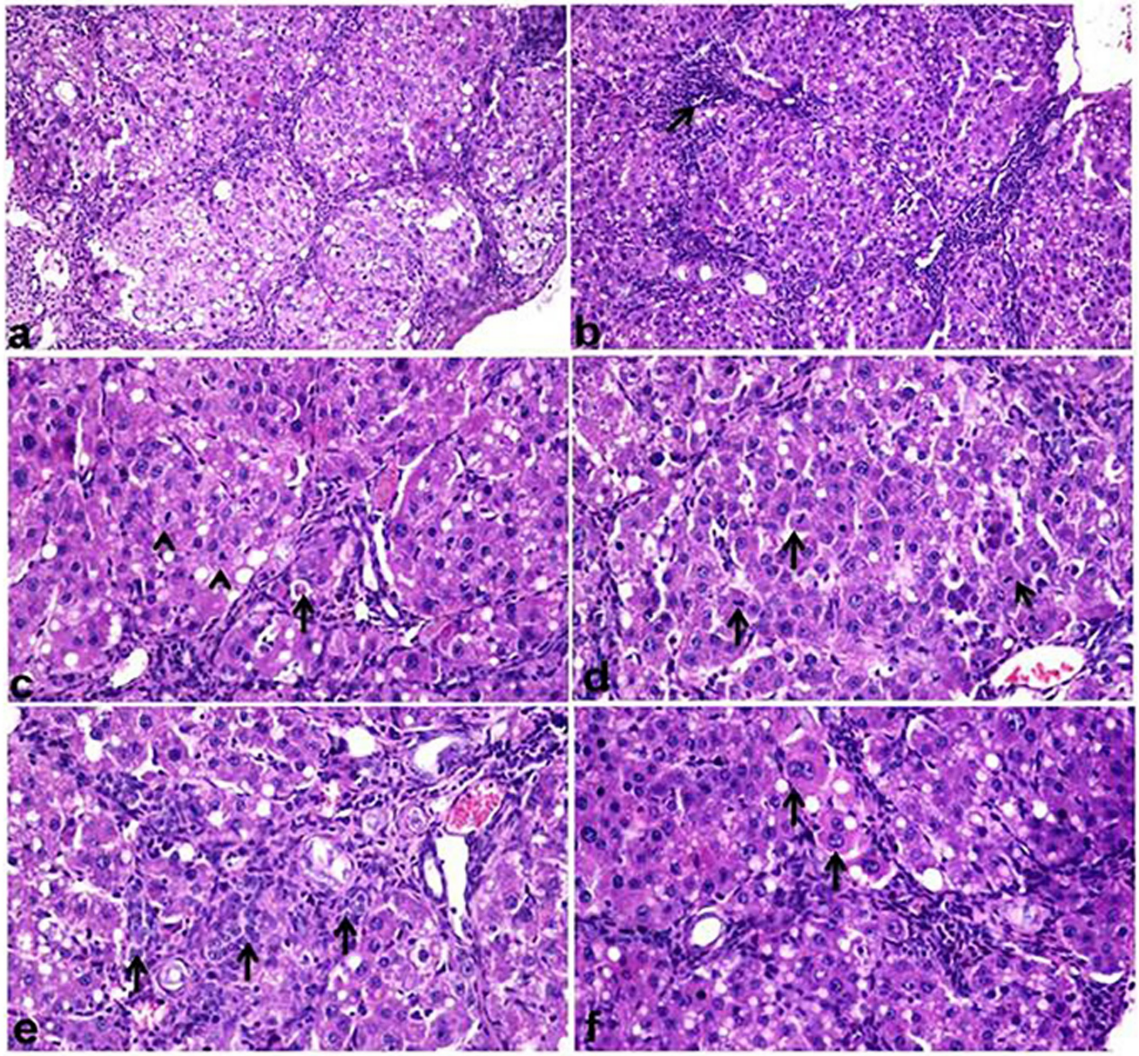
Liver histological sections (H&E) of CCl_4_-intoxicated rat. (a) disruption of hepatic architecture (X200). (b) Mononuclear cell infiltration between hepatic lobules (arrow) consisted mainly of macrophages associated with macrovesicular steatosis of hepatocytes (X200). (c) Apoptosis of individual hepatocytes (arrow) with appearance of mitotic figures(arrow head) (X400). (d) Hepatocytes with various figures of mitosis (arrow) (X400). (e) Oval cell proliferation in the portal area and extending into hepatic lobule (arrow) (X400). (f) Hepatocytes with cytomegaly, binucleation and karyomegaly (arrow) (X400).

Fibrosis grades and intensity of collagen deposition was tabuled in Table 3. The control untreated group showing normal histological structure of hepatic parenchyma. Microscopic examination of CCl_4_ treated group showed various histopatholiogical alterations indicating chronic liver injury and liver fibrosis. Hepatic parenchyma showed disruption of hepatic lobular structure with bridging fibrosis Fig 4A. The hepatocytes showed diffuse macrovesicular steatosis associated with inflammatory reaction Fig 4B, apoptosis with appearance of mitotic figures Fig 4C and 4D were detected. Oval cell proliferation along with bile ductular hyperplasia and kupffer cells activation were evident Fig 4E. Polyploidy of hepatocytes was noticed that was represented by hepatocytomegally, karyomegally, anisokaryosis and increased number of binucleated hepatocytes Fig 4F.

**Table 3:** Histopathological lesions scoring in different treated groups.

Group	Grade of fibrosis	Connective tissue intensity	α- SMA expression	Caspase-3 expression
CCl_4_	3.18 ± 0.88 ^(c)^	30.04 ± 24.31^(b)^	11.48 ± 1.93 ^(b)^	18.88 ± 3.52 ^(b)^
Si+ CCl_4_	1.46 ± 0.63 ^(ab)^	9.29± 1.42 ^(a)^	2.83 ± 1.17 ^(a)^	9.95 ± 1.67 ^(a)^
F-Et+ CCl_4_	1.51 ± 0.70 ^(b)^	10.21 ± 10.08 ^(a)^	2.96 ± 1.21 ^(a)^	11.81 ± 1.29 ^(a)^
B-Et _+_ CCl_4_	1.16 ± 0.65 ^(a)^	6.37 ± 2.10 ^(a)^	2.72 ± 0.57 ^(a)^	8.05 ± 1.39 ^(a)^

Each value expressed as mean ± SEM, (n = 5).

Different letters in the same column are significantly different (p ≤ 0.05).

**Fig 5:**
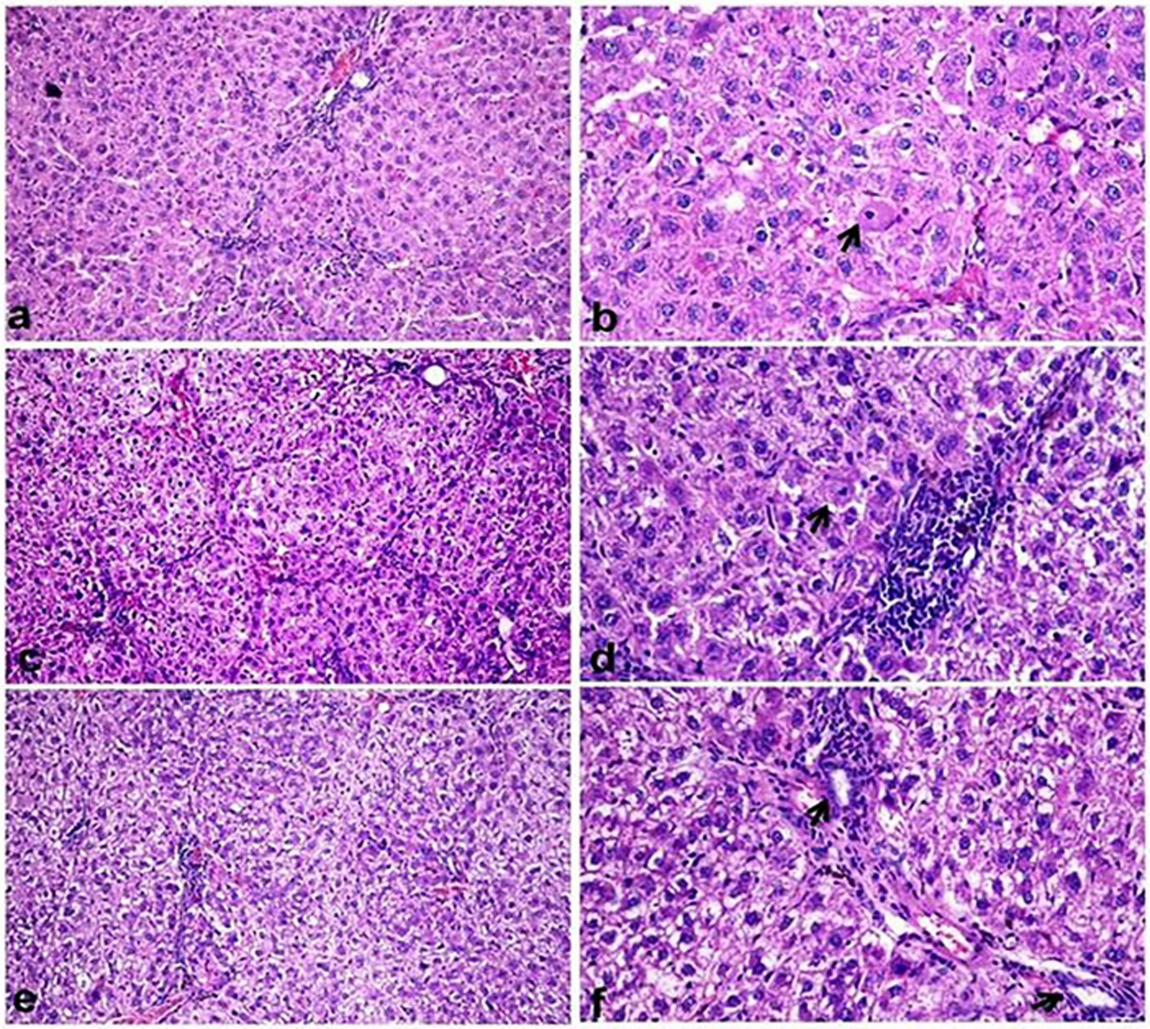
Liver histological sections (H&E). (a) Sylimarin+CCl_4_ showing mild oval cell proliferation in portal area with few mononuclear cell infiltration (X200). (b) kupffer cell activation with individual mitosis of hepatocytes (arrow) and binucleated hepatocytes(X400). (c) F-Et + CCl_4_ showing mild disruption of hepatic lobular architecture with fibrous septa separating hepatic lobules with vacuolization of hepatocellular cytoplasm(X200). (d) mononuclear cell infiltration in the portal area and individual mitosis of hepatocytes (arrow) (X400). (e) B-Et +CCl_4_ showing diffuse vacuolization of hepatocytes with maintaining of lobular strucure(X200). (f) mild mononuclear cell aggreation,oval cell proliferation with newly formed bile ductules (arrow) (X400).

The microscopic examination of group 2 (Si + CCl_4_) revealed reduction in histopathological hepatic alterations that was induced by CCl_4_ treatment. The hepatic lobular architecture was maintained Fig 5A, macrovesicular steatosis of hepatocytes was scarce involving individual hepatocytes with incidental mitosis of hepatocytes Fig 5B associated with moderate kupffer cells activation. Oval cell proliferation was observed in portal area. Group 3 treated with (F-Et + CCl_4_) was more similar to silymarin treated group but the extent of fibrosis was pronounced with more disruption of hepatic architecture Fig 5C. Oval cell proliferation, inflammatory cell infiltration and individual mitosis were detected Fig 5D. The microscopic examination of liver from group 4 that was treated with (B-Et + CCl_4_) revealed minimal hepatic fibrosis Fig 5E with vacuolization of hepatocellular cytoplasm and minimal oval cell proliferation and mild inflammatory cell infiltration Fig 5F.

**Fig 6:**
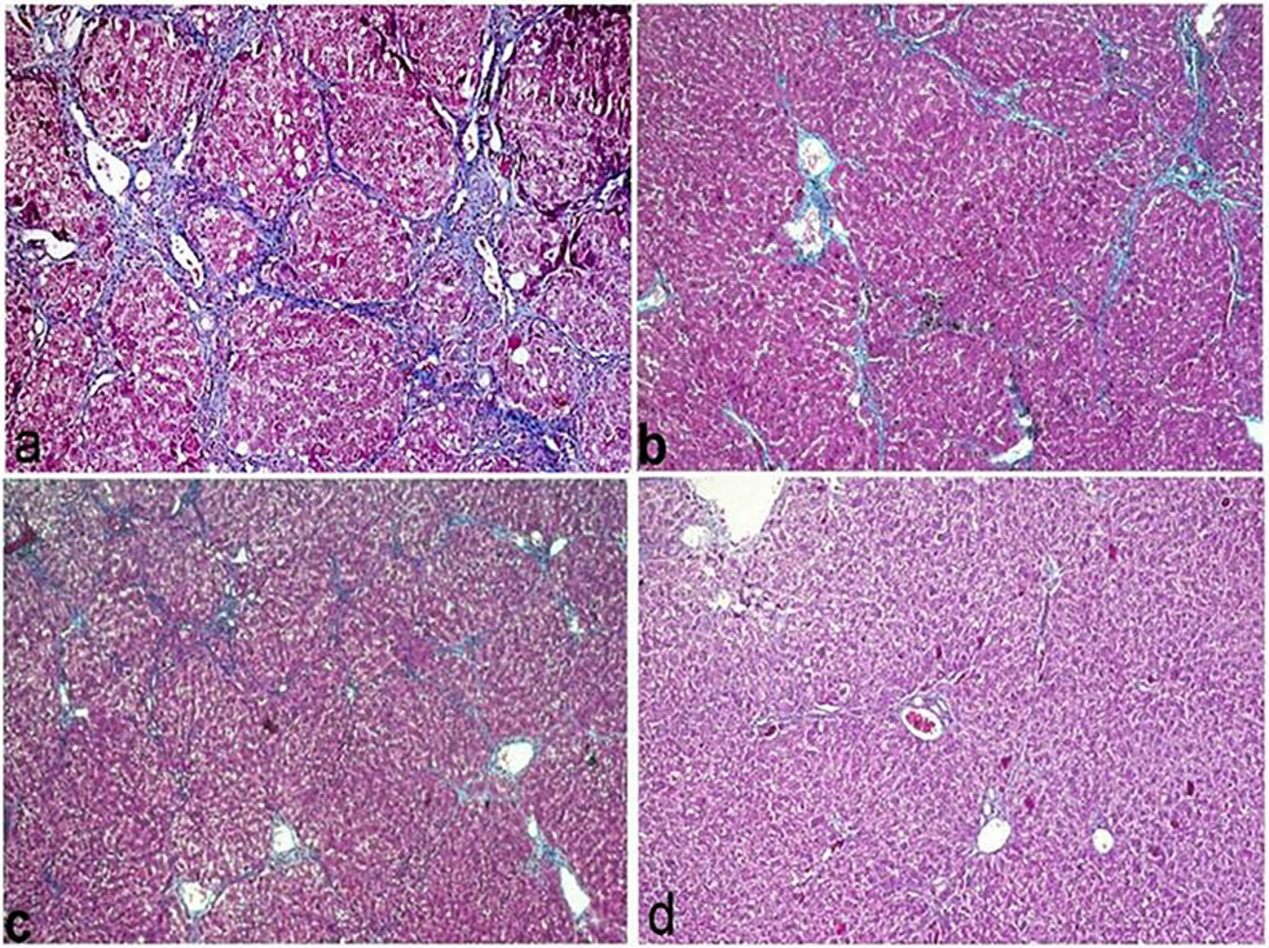
Liver histological sections stained with Masson`s Trichrom. (a) CCl_4_-intoxiated group showing dense bridging fibrosis with pesudolobules formation(X200). (b) sylimarin+ CCl_4_ group showing occasional bridging fibrosis (X200). (c) F-Et+CCl_4_ showing bridging fibrosis (X100). (d) B-Et +CCl_4_ group showing fine fibrous septa extending from portal are into hepatic lobules (X100).

The grade of fibrosis and percentage of collagen deposition in different treated groups are tabuled in Table 3. The histochemical result of CCl_4_ intoxicated group revealed bridging fibrosis with pseudolobules formation, the fibrosis extended from the portal areas into hepatic lobules with significant increase in fibrosis grade and the percent of collagen deposition Fig 6A. However, in group 2 (Si + CCl_4_) the fibrosis grade was reduced significantly compared to CCl_4_ treated group, revealed by mild fibrosis and fine septa extended between hepatic lobules Fig 6B. Similar finding was detected in group 3 (F-Et +CCl_4_) as fibrosis extended as more diffusible fibrous septa from portal area into hepatic lobules compared with the previous group Fig 6C and the least values of fibrosis grade and collagen deposition were recorded in group 4 (B-Et+ CCl_4_) and fibrosis was minimal observed as extremely fine fibrous septa that extended into hepatic lobules Fig 6D.

### Histochemical and immunohistochemical examinations

**Fig 7:**
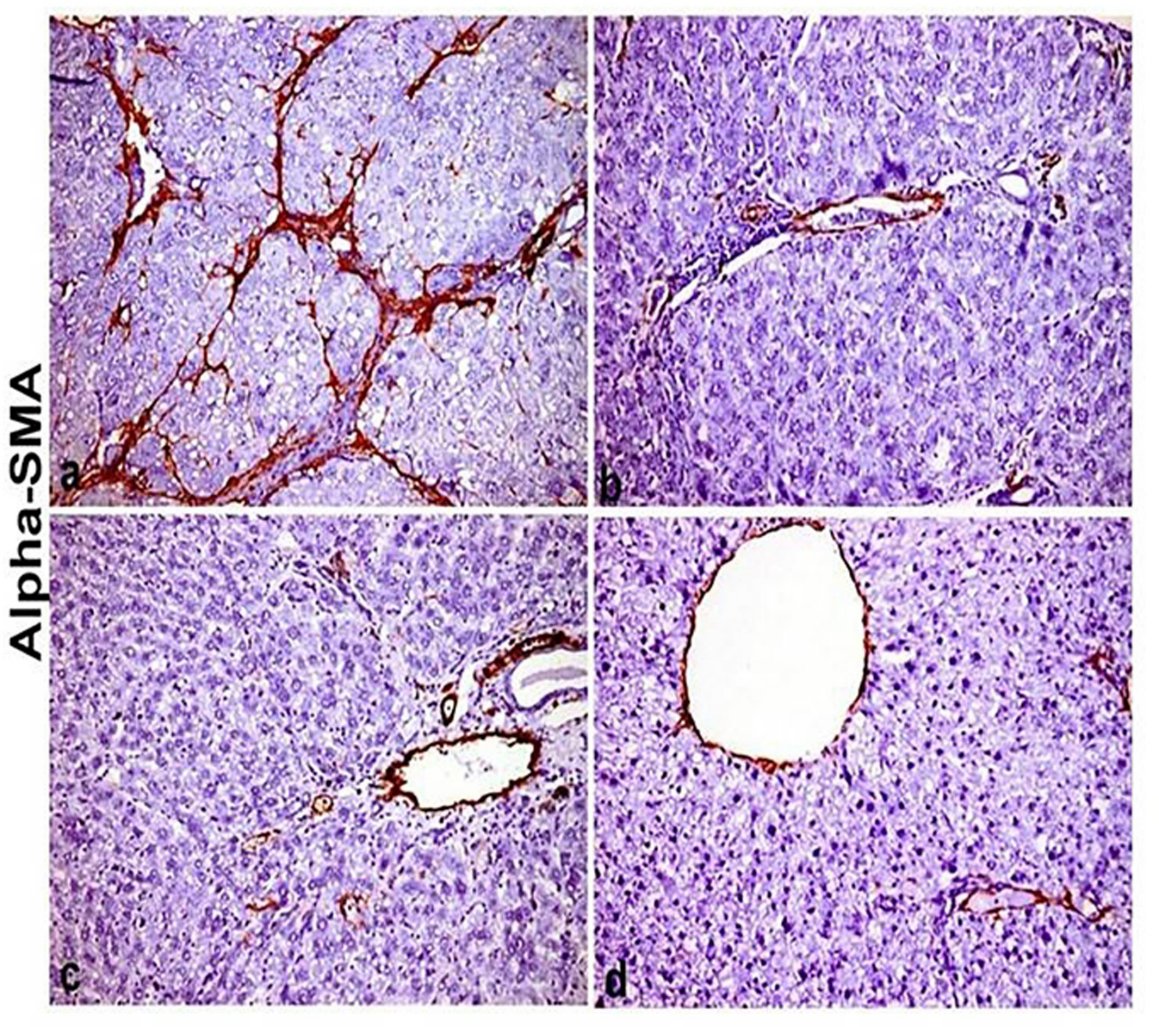
Immunohistochemical stainig of liver section stained with alpha smooth muscle actin antibody(α-SMA) (X200). (a) CCl_4_ intoxicated group showing dense brown expression of α-SMA in between hepatic lobules and into hepatic lobules between hepatocytes. (b) sylimarin+CCl_4_ group showing fine expression of α-SMA in portal area and in between hepatic lobules. (c) F-Et+ CCl_4_ showing more expression of α-SMA into hepatic lobules (X100). (d) B-Et +CCl_4_ group showing faint expression of α-SMA between hepatic lobules.

Immune positive expression of α-SMA was restricted into the smooth muscle wall of portal and central vasculatures of control untreated group. While strong positive α-SMA expression was detected in CCl_4_ treated group, the expression was detected in hepatic stellate cells (HSCs) in the bridging fibrous bands as well as in individual hepatocytes Fig 7A. On other hand, the expression was significantly reduced in sylimarin treated group Fig 7B and the two other treated groups Fig 7C and 7D as shown in Table 3, the expression was restricted to HSCs and not detected in hepatocytes.

**Fig 8:**
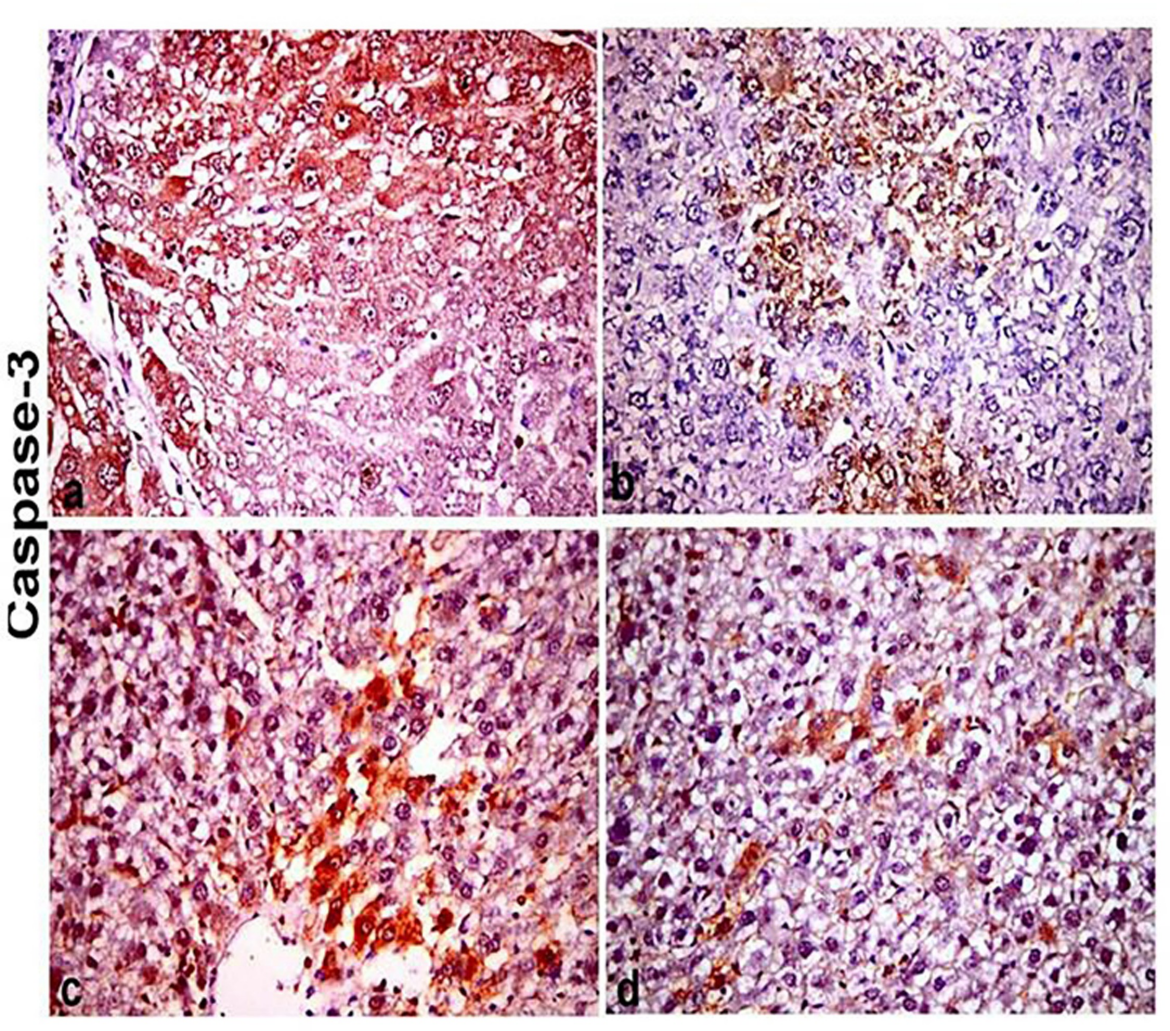
Immunohistochemical stainig of liver section stained with caspase-3 antibody(X400). (a) CCl_4_ intoxicated group showing diffuse and dense cytoplasmic and nuclear caspase-3 expression in hepatocytes. (b) Si+ CCl_4_ group showing focal and faint cytoplamic and nuclear caspase-3 expression in heaptocytes. (c) F-Et+ CCl_4_ group showing focal and less dense cytoplamic and nuclear caspase-3 expression in heaptocytes. (d) B-Et + CCl_4_ group showing individual hepatocytes cytoplamic and nuclear caspase-3 expression.

The expression of caspase-3 was increased in hepatocytes of CCl_4_-itoxicated group and faint expression was observed in silymarin treated group and the two other treated groups. The caspase-3 expression in both hepatocellular cytoplasm and nuclei was strong in CCl_4_- intoxicated group Fig 8A. There was no difference in expression of caspase-3 in hepatocytes between the group 2, 3 and 4 and there was expression in HSCs but not significantly expressed in histological sections Fig 8B, 8C and 8D.

## Discussion

To our knowledge, this current study is the first qualitative profile of *A*. *elaphroxylon* extracts using modern UPLC-Orbitrap-HRMS analysis. Chemical profiling of flowers, bark and leaves methanolic extracts gives insights in the chemical versatility of *A*. *elaphroxylon* extracts as a preliminary step to inaugurate quality assessment of these extracts. Concerning flavonoids, kaempferol glycosides were the major constituents of the three tested extracts. Naringenin glycosides were also recognized in the form of *C-*glycoside for first time in *Aeschynomene* spp.

Triterpenoid saponins were detected for first time in *A*. *elaphroxylon*. These were recognized in both negative and positive ion modes only in flowers and bark extracts. On the contrary, leaves extract was devoid of triterpenoid saponins.

Most of the detected saponins were from soyasaponins; oleanane triterpenoid glycosides having complex and various structures. Soyasaponins are classified according to their individual aglycones (soyasapogenols), with two main aglycones, named as group A and group B respectively [51]. Group B were the major detected soyasaponins. Group B soyasaponins have one glycosylation site on their aglycones (carbon 3) and categorized into two groups, depend on the conjugation at carbon 22 with a 2, 3-dihydro-2, 5-dihydroxy-6-methyl-4-pyrone (DDMP) moiety or without DDMP conjugation. DDMP conjugated soyasaponins are known as *αg*, *βa*, *βg*, *γa* and *γg* while, non-DDMP conjugated soyasaponins are named as soyasaponins I, II, III, IV and V [52]. Soyasaponin V is the major saponin detected in flowers extract, while soyasaponin I is the most abundant saponin in bark extract. One hederagenin saponin was detected only in the bark extract, while pisumsaponin II was present in both flowers and bark extracts.

From the above discussion, it is concluded that both flowers and bark extracts have similar chemical profile mainly for soyasaponins that were identified only in the flowers & bark extracts and were not detected in the leaves extract. Reviewing the available literature these compounds were reported for their hepatoprotective activity by [10,11]. In addition to the antioxidant & hepatoprptective effect of flavonoids; kampferol & quercetin [53]. *In vitro* antioxidant assays for the three extracts have proved that flowers and bark extracts have significant antioxidant activity as compared to leaves extract.Hence, a comprehensive *in vivo* study was designed to verify that this conclusion.

*The in vivo* study publicized the effect of *A*. *elaphroxylon* flowers and bark extracts after 8 weeks of CCl_4_-intoxication in rats. In the present study adult male wistar rats were used as it was found that male wistar rats are more sensitive to CCl_4_ induced hepatotoxicity than female ones [54]. Also, The fibrotic response of the female liver to CCl_4_ treatment was significantly weaker than that of male liver [55]. Administration of CCl_4_ to rats induce hepatocellular toxicity that was expressed by significantly increased serum ALT and AST activities and albumin content. Flowers and bark ethanolic extracts countered these alterations by significantly reducing serum ALT and AST and albumin, as compared to silymarin.

One of the most key mechanisms of CCl_4_-induced hepatotoxicity and progression to fibrosis is the oxidative stress [56]. CCl_4_-induced liver injury often leads to the significant depletion of GSH and accumulation of lipid peroxides in hepatic tissues which are the most important indexes about antioxidant activities in CCl4-induced liver injury. Regarding the effect of flowers and bark extracts, it was found that both extracts significantly reduce lipid peroxidation and increase endogenous antioxidant activity, with a marked increase in hepatic GSH and reduction in the MDA level. The mechanism of liver regeneration in the present study was related to the antioxidant nature of the extract which enable to scavenger the free radicals induced by CCl4, anti-inflammatory effect of the extract that plays a crucial role in initiation of fibrosis and activation of extracellular matrix deposition via hepatic stem progenitor cells proliferation.

With respect to inflammatory processes, it also intensely implicated in the pathogenesis of CCl_4_-induced hepatic fibrosis via the activation of Kupffer cells and release of pro-inflammatory cytokines and adhesion molecule [57]. The results revealed that TNF-α level was significantly reduced by both flowers and bark extracts.

This result was supported by histopathological and immuno-histochemical examinations. In the present work CCl_4_ was chosen instead of paracetamol and acetaminophen as the histopathological picture of paracetamol and acetaminophen-induced hepatotoxicity differ from CCl_4_ in the following; Necrosis is more in paracetamol and acetaminophen than apoptosis while apoptosis was marked in CCl_4_ induced hepatotoxicity, paracetamol and acetaminophen induced weak fibrosis in mice while fibrosis was marked in CCl_4_ in both rats and mice [58]. Various histological degenerative, inflammatory and fibrotic reactions were induced in liver by CCl_4_ treatment in rats. Diffuse steatosis of hepatocytes was observed in CCl_4_ treated group along with apoptosis, anisokaryosis and mitosis of hepatocytes. Researches discussed the mechanism of CCl_4_ induced fat accumulation in liver, CCl_4_ induced hepatic steatosis via reduction of triglycerides execration and hydrolysis from liver, increased their synthesis, disturbed the lipoprotein transport mechanism from liver, in addition CCl_4_ was proved to disturb macromolecules exocytosis as lipoprotein and serum protein from hepatocytes membrane [56]. CCl_4_ induced lipid peroxidation of endoplasmic reticulum membrane with subsequent membrane degradation. The membrane degradation resulted in the release of its PUFA into the cell and formation of malondialdehyde that negatively affect cell membrane. In the present work, apoptosis was induced by CCl_4_ revealed by increase in cellular peroxidation and enhancement of oxidative stress in hepatocytes with subsequent increase in expression of caspase -3 in cytoplasm and nuclei of hepatocytes, Sinha *et al*. [59]. Proved the role of increased peroxidation of hepatocytes in the initiation of apoptosis of kupffer cell activation observed in CCl_4_ treated group and it was reported that it is the enhancement of the production of inflammatory cytokines that initiates the inflammatory reaction in the liver [60]. Mitotic figures were detected in the liver treated with CCl_4_ as there was an evidence that sustained necrosis stimulate proliferation and division of hepatocytes with later developed to cancer. Liver fibrosis resulted to necrosis and inflammation with subsequent oval and stellate cells proliferation and mononuclear cell infiltration. The fibrosis occurred when the necrosis is so severe to overcome the capacity of liver regeneration; the necrotic tissues were replaced by fibrous tissue. Theses histological hepatic changes were the principal alterations induced by CCl_4_ in experimental rodent [61]. Oxidative stress is assumed to initiate and progress the hepatic fibrosis [62]. Our finding evidence that CCl_4_ induced oxidative damage of the hepatocytes with increased MDA activity, steatosis, and inflammatory reaction. These changes were responsible for liver fibrosis as elevation of fibrogenic cytokines by inflammatory reaction in addition to increased proliferation of HSC with subsequent increased expression of α-SMA. The positive correlation was found between increased α-SMA by proliferated HSCs and increased collagen deposition and increased grade of fibrosis. Activated HSCs produced transforming growth factor (TGF-β1) that not only induced collagen production but also enhancement of the tissue inhibitors of metalloproteinase that had role in degradation of extracellular matrix [63]. In the present work, there was a positive correlation between level of TNF-α, the inflammatory reaction of hepatic tissue and degree of fibrosis induced by CCl_4_ toxicity, these positive correlation was discussed by yang *et al*.[64] who reported that TNF-α induced HSC survival, hepatocytes necrosis, and inflammatory reaction all resulted in hepatic fibrosis. Reduction in hepatocellular steatosis, inflammatory reaction and decrease expression of α-SMA by proliferated HSCs by the three therapeutic agents either Si+CCl_4_ or F-Et and B-Et might be the possible mechanism of hepatoprotection against CCl_4_ induced liver fibrosis. Researches confirmed the role of controlling the proliferation of HSCs in treatment of liver fibrosis [65].

## Conclusion

This study ascribes a comprehensive map for the ethanolic extracts of flowers, leaves and bark of *A*. *elaphroxylon* (Guill. & Perr.) Applying UPLC-Orbitrap HRMS fingerprinting method a total of 49 compounds were identified. Where, twelve triterpenoidal saponins were identified for the first time in the plant, saponins were detected only in flowers & bark extracts, in addition to phenolic compounds. These identified compounds rationalize the prophylactic effect of the extracts against CCl_4_ induced toxicity. It should be mentioned that this result should be further supported by preclinical/ clinical studies to permit their use as new hepatoprotective drugs.
